# MicroRNAs and angiosarcoma: are there promising reports?

**DOI:** 10.3389/fonc.2024.1385632

**Published:** 2024-05-17

**Authors:** Amir Modarresi Chahardehi, Arya Afrooghe, Nikoo Emtiazi, Sajjad Rafiei, Negin Jafarkhanloo Rezaei, Sarvin Dahmardeh, Fatemeh Farz, Zahra Naderi, Reza Arefnezhad, Hossein Motedayyen

**Affiliations:** ^1^ Kimia Andisheh Teb Medical and Molecular Research Laboratory Co., Tehran, Iran; ^2^ School of Medicine, Tehran University of Medical Sciences, Tehran, Iran; ^3^ Department of Pathology, Firoozgar Hospital, Iran University of Medical Sciences, Tehran, Iran; ^4^ Medical Toxicology and Drug Abuse Research Center (MTDRC), Birjand University of Medical Sciences, Birjand, Iran; ^5^ School of Medicine, Najafabad University of Medical Sciences, Isfahan, Iran; ^6^ Faculty of Medicine, Mashhad University of Medical Sciences, Mashhad, Iran; ^7^ Student Research Committee, Ahvaz Jundishapur University of Medical Sciences, Ahvaz, Iran; ^8^ School of Medicine, Ahvaz Jundishapur University of Medical Sciences, Ahvaz, Iran; ^9^ Coenzyme R Research Institute, Tehran, Iran; ^10^ Student Research Committee, Shiraz University of Medical Sciences, Shiraz, Iran; ^11^ Autoimmune Diseases Research Center, Kashan University of Medical Sciences, Kashan, Iran

**Keywords:** microRNA, angiosarcoma, cancer, cardiovascular tumor, tumor suppressor, oncogenesis

## Abstract

In recent years, microRNAs (miRNAs) have garnered increasing attention for their potential implications in cancer pathogenesis, functioning either as oncogenes or tumor suppressors. Notably, angiosarcoma, along with various other cardiovascular tumors such as lipomas, rhabdomyomas, hemangiomas, and myxomas, has shown variations in the expression of specific miRNA subtypes. A substantial body of evidence underscores the pivotal involvement of miRNAs in the genesis of angiosarcoma and certain cardiovascular tumors. This review aims to delve into the current literature on miRNAs and their prospective applications in cardiovascular malignancies, with a specific focus on angiosarcoma. It comprehensively covers diagnostic methods, prognostic evaluations, and potential treatments while providing a recapitulation of angiosarcoma’s risk factors and molecular pathogenesis, with an emphasis on the role of miRNAs. These insights can serve as the groundwork for designing randomized control trials, ultimately facilitating the translation of these findings into clinical applications. Moving forward, it is imperative for studies to thoroughly scrutinize the advantages and disadvantages of miRNAs compared to current diagnostic and prognostic approaches in angiosarcoma and other cardiovascular tumors. Closing these knowledge gaps will be crucial for harnessing the full potential of miRNAs in the realm of angiosarcoma and cardiovascular tumor research.

## 1 Introduction

MicroRNAs (miRNAs) are a class of small, non-coding, single-stranded RNAs that regulate gene expression at the post-transcriptional level, impacting crucial cellular processes like proliferation, differentiation, and apoptosis (1–4). This regulatory influence extends to an estimated 60% of human genes, highlighting their widespread impact (5). Consequently, miRNAs are attracting significant interest as potential targets for the prevention, diagnosis, and treatment of complex human diseases (6–8). Emerging evidence underscores the critical role of miRNAs in maintaining cellular homeostasis by tightly regulating diverse biological functions (9, 10). Notably, miRNAs can function as either oncogenes or tumor suppressors, playing a pivotal role in cancer biology (11–13). Dysregulation of miRNA expression is frequently observed in various types of cancer (12, 14). These alterations contribute to cancer hallmarks by enhancing proliferation signals, evading growth inhibitors, resisting cell death, promoting invasiveness, and stimulating angiogenesis (15). The profound influence of miRNAs on these crucial cancer-related processes suggests their immense potential for future research and therapeutic development.

Angiosarcoma is a rare and aggressive soft-tissue sarcoma characterized by a high propensity for local recurrence, distant metastasis, and poor prognosis if not diagnosed early (16). Endothelial cells lining blood and lymphatic vessels give rise to angiosarcoma, contributing to its frequent lymph node and systemic metastases (17, 18). While primarily affecting the skin and soft tissues, particularly the head and neck region, angiosarcoma also represents the most common form of differentiated cardiac malignancy, accounting for approximately 10–15% of primary cardiac tumors (16, 17, 19). Despite advancements in cancer therapy, angiosarcoma management remains challenging, highlighting the need for a deeper understanding of its molecular underpinnings and the development of novel therapeutic strategies (18). The rarity of this disease translates to a lack of established treatment protocols. While localized surgery offers the best chance of cure, technical challenges can limit its feasibility in some cases (20). Emerging evidence suggests that various microRNA subtypes might influence angiosarcoma development and progression (21). For instance, tumor cells often exhibit upregulation of miR-210, leading to the downregulation of its putative targets, ephrin A3 and E2F3 (22). Additionally, miRNAs hold promise as potential diagnostic biomarkers for angiosarcoma. Exosomal miR-5684 and miR-125b-5p are significantly downregulated in angiosarcoma patients compared to healthy controls (23). Furthermore, dysregulation of miRNAs like miR-222, miR-199a, miR-195, and miR-125b has been observed in angiosarcoma, potentially influencing cell cycle proteins and contributing to cell cycle deregulation (24). Thus, targeting these aberrantly expressed miRNAs represents a promising therapeutic approach for improved treatment outcomes and survival rates in angiosarcoma patients.

Given the emerging recognition that microRNAs (miRNAs) exhibit differential expression patterns across various cancer types, grades, and clinical courses, these molecules hold significant promise as diagnostic, prognostic, and even therapeutic tools (15). Therefore, this review delves into angiosarcoma, a rare and aggressive soft-tissue sarcoma. We will first provide a concise overview of angiosarcoma risk factors and molecular pathogenesis. Following this foundation, we will critically evaluate the current evidence regarding the role of miRNAs in angiosarcoma. Our focus will be on the potential of miRNAs as future targets for angiosarcoma diagnosis, prognosis, and therapy. Finally, we will broaden the scope to discuss the potential significance of miRNAs in other cardiovascular tumors.

## 2 Risk factors and molecular pathogenesis of angiosarcoma

Angiosarcoma is an uncommon and aggressive soft tissue sarcoma, accounting for 1-2% of all diagnosed sarcomas (25). This section presents a comprehensive summary of the occurrence, development, diagnosis, prediction of outcomes, survival rates, and treatment choices for angiosarcoma. Angiosarcomas can manifest at any age but are predominantly detected in older individuals, with a median age of onset ranging from 60 to 71 years (25). The annual prevalence is approximately 1-2 cases per million individuals (26), with an incidence rate adjusted for age ranging from 1-4% (27).

The development of angiosarcoma is intricate and needs to be completely comprehended. Nevertheless, certain variables that increase the risk of angiosarcoma have been identified, including long-term lymphedema, previous radiation therapy, and exposure to environmental substances that might cause cancer (28). Evidence suggests that genetic predispositions, such as POT1 gene mutations, play a role in familial angiosarcoma cases (29). Diagnosis of angiosarcoma might be challenging due to its infrequency and lack of identifiable clinical symptoms. Diagnostic procedures such as ultrasound, CT, and MRI visualize internal structures. However, a conclusive diagnosis involves examining tissue samples under a microscope and confirming with immunohistochemistry tests. Markers such as CD31, CD34, and Factor-VIII indicate the origin of vascular endothelial cells (25). Prognostication is influenced by several parameters, including the size, location, resectability, and existence of tumor metastases at the time of diagnosis (28). However, the survival rates for angiosarcoma exhibit significant variability. The reported total 5-year survival rate ranges from 27% to 40%, with median survival durations varying from 6 to 16 months (27). Patients diagnosed with high-grade tumors or those who already have metastasized illness upon presentation have a meager chance of a good prognosis (25). Surgical excision, frequently accompanied by adjuvant radiation, is the cornerstone of treatment for localized angiosarcoma (25). Chemotherapy, using anthracycline or taxane-based regimens, is commonly employed to treat instances that cannot be operated on or cases that have spread to other body parts. Current advancements in targeted treatments and immunotherapy are potential therapeutic alternatives, while the specific order and combination with traditional medications are still actively studied (30).

### 2.1 Risk factors

**Figure 1:**
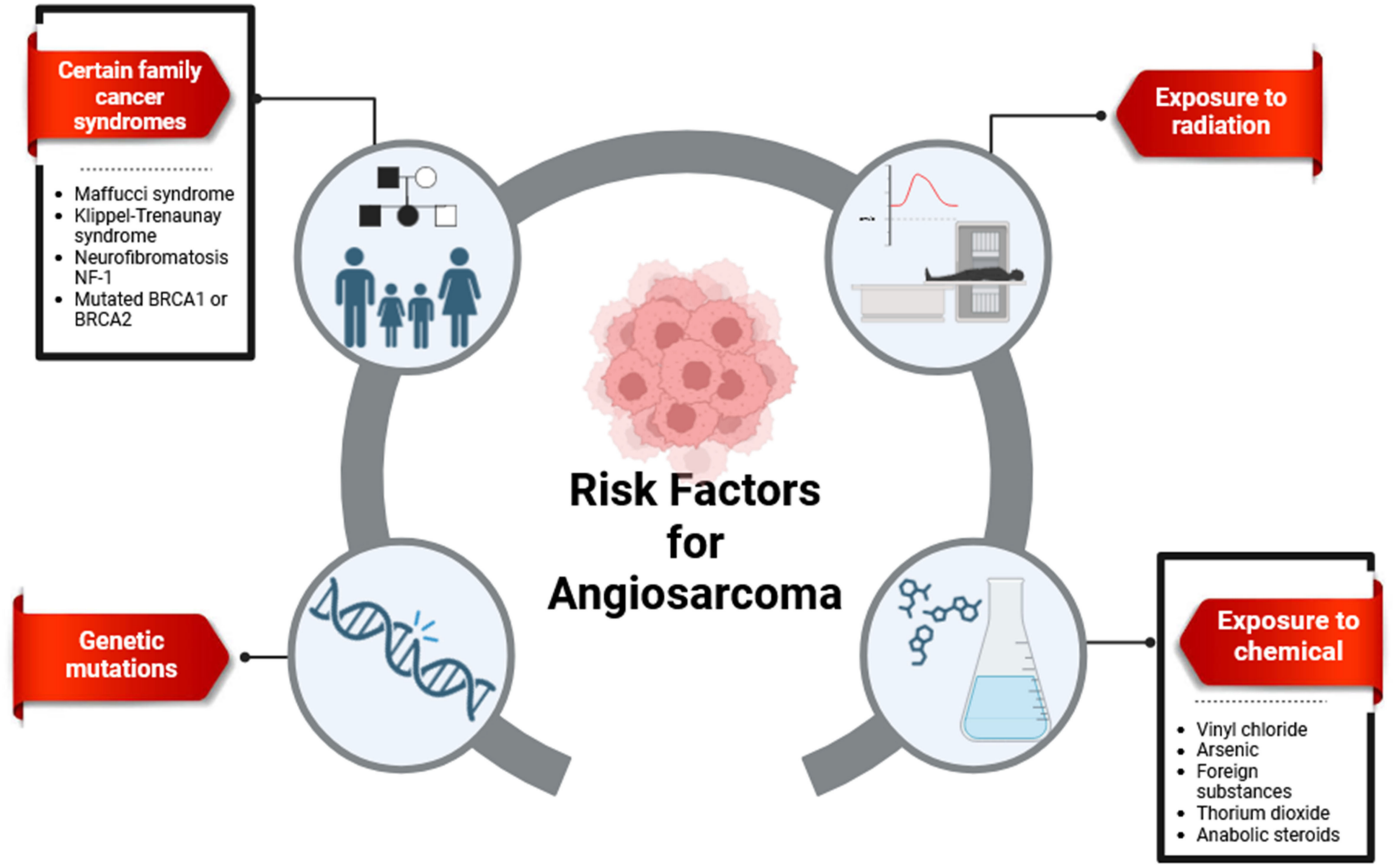
Several risk factors for angiosarcoma (adapted from Ni et al. (33) (33).

The etiology of angiosarcoma remains unclear; however, several well-established risk factors have been identified. These include exposure to radiation, chronic lymphedema, and carcinogens such as thorium dioxide, vinyl chloride, and arsenic. Genetic disorders also contribute to the constellation of risk factors for angiosarcoma (31, 32), as illustrated in **Figure 1**.

#### 2.1.1 Radiation

The link between radiation exposure and increased cancer risk is well-established. Chronic lymphedema and radiation-induced genetic changes are potential contributing factors to radiation-associated malignancies (34, 35). Notably, radiation therapy for early-stage sarcomas can paradoxically lead to radiation-induced sarcomas, a significant subtype of secondary sarcomas (36, 37). While the exact link between radiation and angiosarcoma development needs further investigation, studies suggest a potential association between higher radiation doses, longer lifespans after treatment, and the risk of radiation-induced angiosarcoma (38, 39). Previous radiation exposure might also be a risk factor (40). However, the overall risk of radiation-induced angiosarcoma remains low when balanced against the benefits of radiotherapy (25).

#### 2.1.2 Chronic lymphedema

A well-documented association exists between angiosarcoma and long-term chronic lymphedema, a condition known as Stewart-Treves syndrome (STS) (41). STS predominantly affects women, particularly following adjuvant radiation and breast surgery (41, 42). The use of adjuvant radiation during treatment for the primary malignancy is believed to contribute to STS progression (43). Other potential causes of lymphedema that might elevate angiosarcoma risk include idiopathic, congenital, traumatic, or filarial etiologies (44). Approximately 5% of angiosarcomas are attributed to STS, typically presenting within 5-15 years after surgery and radiation (43). Unfortunately, STS is associated with a poor prognosis, with a median expected survival of around ten months (45). The mechanisms underlying the progression from different forms of persistent lymphedema to secondary angiosarcomas remain poorly understood. Hypotheses suggest that mutations in tumor suppressor genes like MYC or P53 might play a co-factor role (45, 46).

#### 2.1.3 Environmental carcinogens

Despite the unclear etiology of approximately 75% of hepatic angiosarcomas (47), exposure to specific environmental carcinogens has been implicated as a potential risk factor. These carcinogens include vinyl chloride monomer (VCM), a component in polyvinyl chloride production, iatrogenic exposure to the radioactive contrast agent Thorotrast used in radiological examinations, and chronic arsenic consumption (48, 49).

#### 2.1.4 Genetic disorders

Approximately 3% of primary angiosarcomas are classified as subtypes associated with familial and genetic disorders. Klippel-Trenaunay syndrome (KTS) is a notable example, characterized by congenital malformations involving blood vessels, soft tissues, and bones. Other genetic disorders linked to angiosarcoma risk include Ollier disease and Recklinghausen neurofibromatosis. Interestingly, these familial predispositions are not specific to angiosarcoma and have also been associated with Xeroderma pigmentosa, Maffucci disease, and bilateral retinoblastoma (50).

### 2.2 Molecular pathogenesis

Angiosarcoma pathogenesis is a complex process driven by a multitude of genetic and epigenetic alterations. Expanding upon the groundwork established in Section 2.1, this section provides a more comprehensive analysis of the precise molecular mechanisms that govern the development of angiosarcoma. Recent studies have brought attention to the intricate relationship that exists among epigenetic dysregulation, mutations in genes, and signaling pathways in the development of angiosarcoma.

#### 2.2.1 Dysregulation of angiogenic pathways and tumor suppressors

Angiosarcoma exhibits significant molecular heterogeneity (31, 51). Notably, studies have reported high-level amplification at the 8q24.21 chromosomal locus and significant MYC overexpression in secondary cutaneous angiosarcomas (52). These findings highlight the importance of reliable methods for MYC detection, such as fluorescent *in situ* hybridization (FISH) or immunohistochemistry, to differentiate cutaneous angiosarcomas from atypical vascular lesions, which are typically MYC-negative (53).

Recent research sheds light on the role of upstream signaling pathways in MYC overexpression. MYC-positive cutaneous angiosarcomas demonstrate overexpression of atypical Protein Kinase C lambda/iota (aPKCλ). Beyond its established functions in cell polarity and cancer progression, aPKCλ appears to influence endothelial cell proliferation in angiosarcoma. Mechanistically, aPKCλ phosphorylates the endothelial transcription factor FoxO1 at Ser218, leading to its inactivation and preventing its binding to the miR-34c promoter. miR-34c normally acts as a negative regulator of MYC expression; thus, its downregulation by aPKCλ-mediated FoxO1 inactivation contributes to MYC overexpression (54).

Interestingly, aPKCλ can upregulate PD-L1 expression in tumor cells, potentially influencing the trajectory of cancerous cells (55). Selective targeting of aPKCλ with specific inhibitors alongside miR-34c mimics warrants further investigation for their therapeutic potential (18).

#### 2.2.2 Mutation landscape and signaling pathway aberrations

Angiosarcoma development is also characterized by distinct mutation patterns in primary and secondary cutaneous subtypes. Secondary cutaneous angiosarcomas frequently harbor mutations in genes like KIT, ICOS, FLT4, and RASGRP3, while primary cutaneous angiosarcomas exhibit mutations in TP53, KRAS, and BRAF among others. Interestingly, primary cutaneous angiosarcomas are also associated with mutations in fusion genes like NUP160-SCL43A3 and PTPRB/VE-PTP (56, 57).

Primary cutaneous angiosarcoma is frequently associated with TP53 loss-of-function mutations. The p53 signaling pathway, activated by various cellular stresses, plays a critical role in tumor suppression (58). Given its function in cell cycle regulation, *p53* mutations are prevalent across diverse cancers, including angiosarcoma (59). Notably, *p53* inhibits angiogenesis by downregulating vascular endothelial growth factor (VEGF). However, Mouse Double Minute 2 Homolog (MDM2) can negatively regulate *p53* activity, leading to increased VEGF expression and promoting angiosarcoma formation (60, 61). Consequently, the presence of anti-p53 antibodies in serum might serve as a potential biomarker for angiosarcoma diagnosis or prognosis (62).

The MAPK signaling pathway is implicated in the pathogenesis of cutaneous angiosarcoma. Dai et al. reported that BRAF inhibitor therapy, while effective in some cancers, can paradoxically activate the MAPK pathway and lead to secondary malignancies like RET-mutant cutaneous angiosarcomas (63). Furthermore, Murali et al. (56) identified frequent mutations in CDKN2A (26%) and TP53 (35%), both affecting the MAPK pathway, suggesting its potential as a therapeutic target. Given the high prevalence of these mutations, specific inhibitors targeting the MAPK pathway might offer a promising treatment approach for aggressive angiosarcoma subtypes (56).

#### 2.2.3 Epigenetic dysregulation

Epigenetic changes, such as adjustments in DNA methylation patterns and histone modifications, are being increasingly acknowledged as significant determinants in the genesis of cancer (64). DNA methylation is an epigenetic alteration that has a significant correlation with the regulation of gene expression. The significance of changes in DNA methylation in the development of tumors motivates our efforts to unravel the human epigenome (65). Previous research on DNA methylation in angiosarcoma has focused solely on the tumor suppressor gene cyclin-dependent kinase inhibitor 2A (CDKN2A, or INK4A/ARF). This has shown that most sporadic liver angiosarcoma cases have hypermethylation of the CDKN2A (p16INK4A) promoter (66). The majority of radiation-associated angiosarcomas, according to Mentzel et al., have lost H3K27me3 expression; however, endothelial cells in benign and atypical vascular lesions that have formed after prior radiation therapy stained positively for H3K27me3. When tested for H3K27me3, the sporadic angiosarcomas showed inconsistent staining. One other diagnostic tool that may be used to identify radiation-associated angiosarcomas is the loss of H3K27me3 (67). While research on epigenetic modifications in angiosarcoma is ongoing, some studies suggest their potential role. For instance, Sirtuins (SIRT1-7), a class of NAD+-dependent deacetylases, have emerged as contributors to various age-related pathologies like cancer, neurodegeneration, and metabolic disorders (68, 69). Interestingly, angiosarcoma cells exhibit decreased SIRT7 expression, which correlates with suppressed cell proliferation and invasion. Furthermore, SIRT7 can counteract the tumor suppressive effects of miR-340, highlighting its complex role in angiosarcoma (21).

#### 2.2.4 Other molecular players in angiosarcoma pathogenesis

Beyond the established roles of angiogenic pathways and tumor suppressor genes, several other molecular players contribute to angiosarcoma development. The E2F family of transcription factors involved in cell cycle progression, survival, and differentiation, are also implicated in angiosarcoma pathogenesis. This highlights the intricate network of molecular pathways involved in this malignancy (70).

Protein tyrosine phosphatase receptor type B (PTPRB) encodes VE-PTP, a phosphatase that inhibits vascular endothelial growth factor receptors (VEGFR) 1-3 in endothelial cells. Given the established overexpression of VEGFRs in angiosarcomas (71, 72), targeting VE-PTP represents a promising therapeutic strategy. Furthermore, amplification of the FLT4 gene, encoding VEGFR3 (73), has been observed in approximately 25% of secondary angiosarcomas alongside MYC amplification, leading to increased FLT4 mRNA expression (74). These findings suggest that targeting FLT4 might be another potential avenue for angiosarcoma treatment.

ATRX loss and reduced expression of Notch1 and Notch2 have also been documented in angiosarcomas. Interestingly, reduced Notch1 expression is associated with advanced disease and cutaneous origin, while lower Notch2 levels correlate with poorer disease-specific outcomes. Moreover, studies suggest a suppressive role for Notch1 in liver endothelial cell function, potentially contributing to hepatic angiosarcoma development (75). Furthermore, the suppression of Notch1 in liver endothelial cells contributed to the development of hepatic angiosarcomas (76).

## 3 MicroRNAs and angiosarcoma

MicroRNAs, also known as miRNAs, are a type of tiny RNA molecules that play a crucial role in regulating gene expression by specifically targeting messenger RNAs (mRNAs) (77). MiRNAs control physiological functions, including cell necrosis, apoptosis, and active secretion through exosome microvesicles (78). The deregulation of miRNA expression is a common observation in tumorigenesis, as miRNA loci are often linked to chromosomal regions containing DNA amplifications, deletions, or translocations (79). Emerging evidence suggests a complex interplay between miRNAs and angiosarcoma. KCa3.1, a calcium-activated potassium channel, regulates endothelial proliferation and angiogenesis. Its expression is significantly upregulated upon repression of miR-497-3p. *In vivo* studies using xenotransplant models demonstrated that TRAM-34, KCa3.1 inhibitor, or miR-497-5p mimetics could impair the growth of angiosarcoma cell lines [59]. Furthermore, biallelic deletion of Dicer1, a crucial enzyme for miRNA biogenesis, has been associated with the development of highly aggressive angiosarcomas [60]. This observation highlights the potential of utilizing antagonistic miRNAs or miRNA mimetics for angiosarcoma therapy [8]. Garcia-Heredia et al. examined miRNA expression in 17 sarcomas and 6 nontumor mesenchymal tissues and compared them to the IMR90 cell line to find differentially expressed miRNAs. They discovered 81 miRNAs (23 down, 49 up) (19).

Accordingly, antagonistic miRNAs or miRNA mimetics, with a specific application toward angiosarcoma, could prove valuable in angiosarcoma therapy in the future (18). The intricate interplay between miRNAs and cancer cells in angiosarcoma, with the potential for therapeutic interventions and diagnostic or prognostic markers, will be discussed in the next section in detail. The characteristics of miRNAs exhibited in cancers may vary depending on the tumor tissue, as these molecules possess both oncogenic and tumor-suppressive properties (80) as follows:

### 3.1 Tumor suppressor

While miRNA deregulation is a common feature in cancers, some miRNAs exhibit tumor-suppressive properties in angiosarcoma (81, 82). miR-214, for instance, acts as a tumor suppressor by targeting the COP1-p53 pathway, thereby restoring the activity of the p53 tumor suppressor gene and promoting programmed cell death in angiosarcoma cells, restoring the activity of p53, a crucial gene that inhibits tumor growth. These findings indicate that targeting the miR-214-COP1-p53 axis is a promising and innovative approach for treating canine hemangiosarcoma and human angiosarcoma (83). Similarly, miR-340 has been shown to inhibit the proliferation and infiltration of angiosarcoma cells by targeting SIRT7, further highlighting its potential as a tumor suppressor miRNA in this aggressive malignancy (21). Reduced cell migration and tumor development were additional outcomes of miR-497 overexpression. An angiosarcoma cell line’s RNA-sequencing data, expression data from AS patients, and target prediction algorithms were utilized to identify therapeutically significant target genes, allowing us to investigate the tumor suppression mechanism of miR-497 (82).

### 3.2 Oncogenic

**Figure 2:**
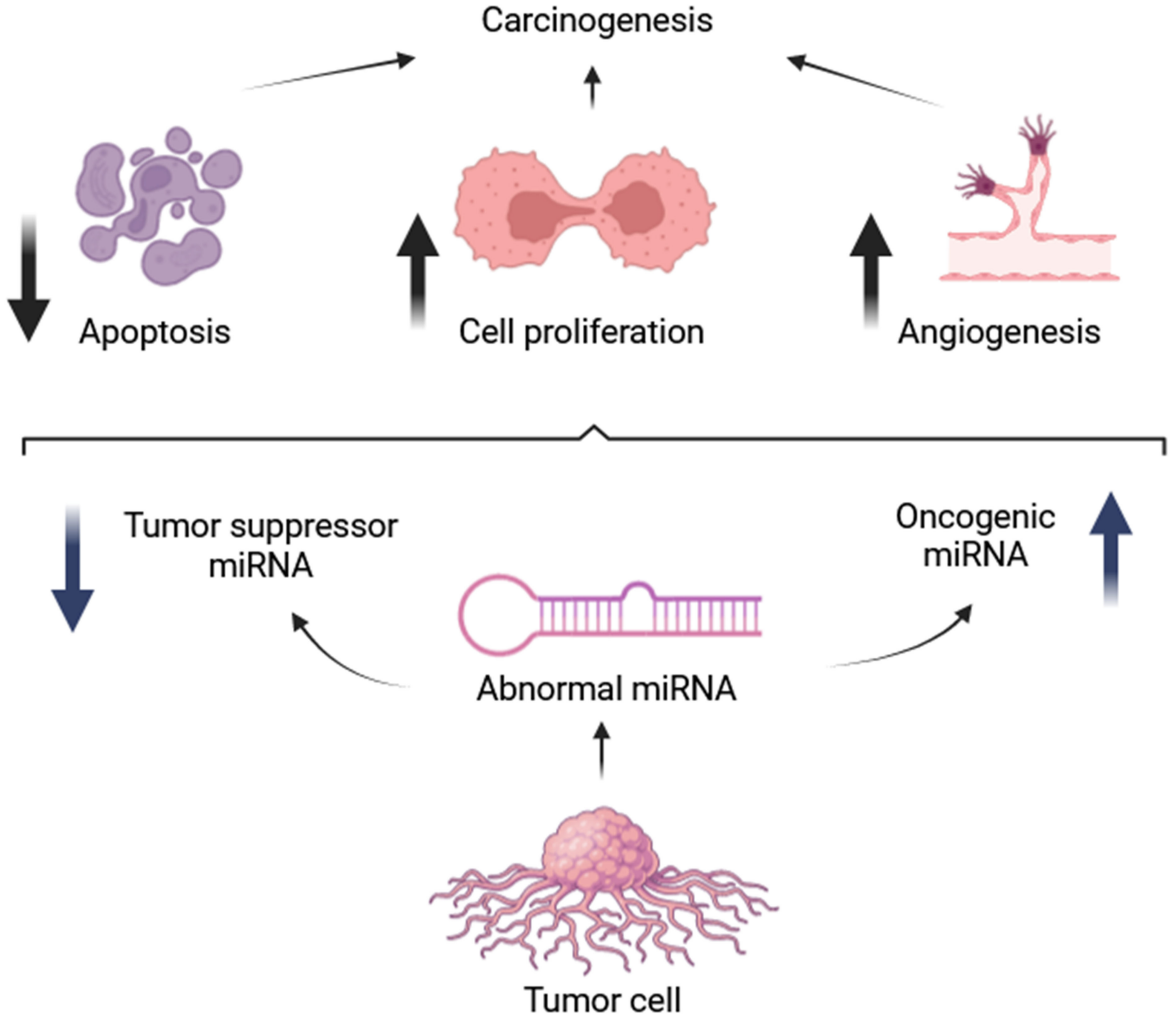
Roles of oncogenic and tumor suppressive microRNAs [adapted from Hata et al. (2015)] (92).

Angiosarcomas, like many cancers, exhibit dysregulation of miRNAs, which can function as both oncogenes and tumor suppressors (84). This characteristic opens avenues for exploring miRNAs as therapeutic targets. Strategies include mimicking tumor-suppressive miRNAs or inhibiting oncogenic miRNAs to manipulate their influence on cancer development (85–87). For example, miR-210 has been shown to promote angiosarcoma cell proliferation by targeting ephrin A3 and E2F3, proteins involved in cell growth and division. Studies revealed upregulation of these target proteins alongside miR-210 overexpression in tumor cells, while knockdown of these targets significantly reduced angiosarcoma cell numbers, suggesting an oncogenic role for miR-210 in this context (22). Interestingly, miR-340 appears to possess a dual role in tumorigenesis, acting as both a tumor suppressor and an oncogene depending on the cellular context (21). Angiosarcoma is frequently associated with mutations in Dicer1, a ribonuclease enzyme essential for processing precursor miRNAs (pre-miRNAs) into mature functional miRNAs, and also has been implicated in angiosarcoma pathogenesis (88, 89). Studies demonstrated that biallelic Dicer1 deletion can lead to aggressive and metastatic angiosarcoma, even in the absence of additional oncogenic mutations (90). Studies in mice have demonstrated that biallelic loss of Dicer1 predisposes endothelial cells to angiosarcoma formation, highlighting the critical role of DICER1 in maintaining proper miRNA biogenesis (90, 91). This finding strengthens the link between miRNA alterations and angiosarcoma development (82). Furthermore, Hanna et al. reported that mice with Dicer1-deleted angiosarcomas exhibit overexpression of target genes for miR-23, a miRNA known to regulate cell cycle progression. The increased expression of miR-23 promotes S phase entry by enhancing the activity of cell cycle regulators CCND1 and CDK4/6 (90). This finding highlights the crucial role of miRNAs in preventing angiosarcoma and suggests that disruption of this regulatory mechanism can promote tumorigenesis (90). In summary, miRNAs act primarily as negative regulators of gene expression. However, excessive levels of oncogenic miRNAs can promote tumor development by stimulating cell proliferation and inhibiting apoptosis, as illustrated in **Figure 2** (93).

**Figure 3:**
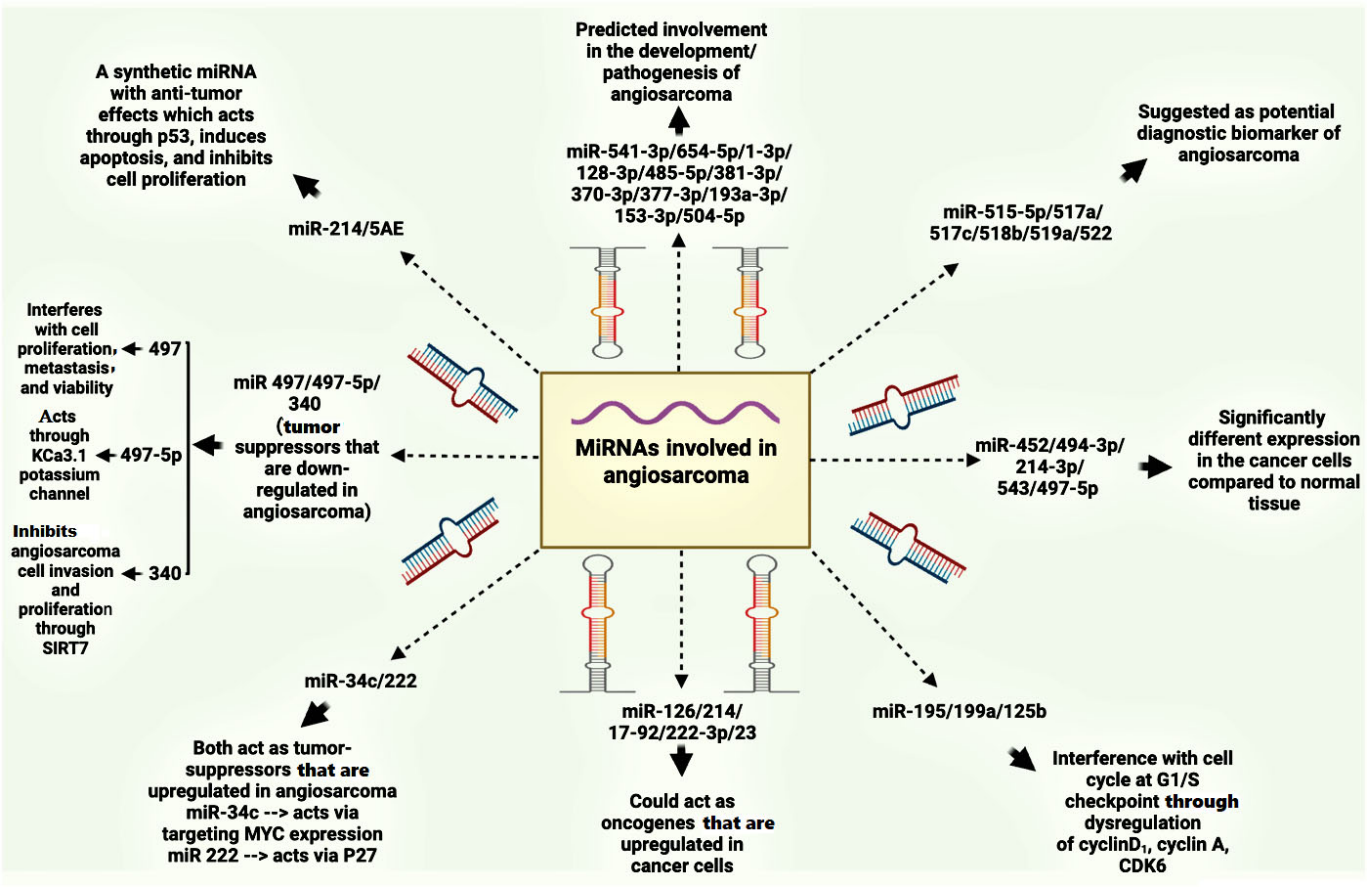
MiRNAs involved in angiosarcoma.

The expanding role of miRNAs in cancer diagnosis, prognosis, and therapy has spurred research into their potential applications in angiosarcoma (94). Several miRNAs have emerged as candidates for angiosarcoma biomarkers. Upregulation of miR-541-3p/miR-654-5p, miR-1-3p, miR-128-3p, miR-485-5p, miR-381-3p, miR-370-3p, miR-377-3p, miR-193a-3p, miR-153-3p, and miR-504-5p (95). Additionally, miRNAs such as miR-515-5p, miR-515-3p, miR-517a, miR-517c, miR-518b, miR-519a, and miR-522 have been suggested as potential diagnostic biomarkers for angiosarcoma due to their overexpression in angiosarcomas (96). These findings are depicted in **Figure 3**.

Heishima et al. reported downregulation of miR-214 and overexpression of COP1 in canine hemangiosarcoma, a model for human angiosarcoma [71]. In this context, miR-214 functions as a tumor suppressor by restoring p53 activity and promoting apoptosis, thereby inhibiting invasion and migration (83, 97). However, studies have revealed unexpectedly high plasma levels of miR-214 and miR-126 in human angiosarcoma patients [83]. Intriguingly, miR-214 is hypothesized to possess anti-angiogenic properties within cells but exert angiogenic effects when released extracellularly, suggesting the involvement of target cells and co-factors like microvesicle proteins in its activity (98). This complex interplay highlights the potential of circulating miR-214 as a precise diagnostic biomarker for tumors, including angiosarcoma (99).

Several reveals altered expression of several miRNAs in the serum of dogs with splenic masses compared to healthy controls. Interestingly, miR-214 levels are specifically elevated in angiosarcoma tissues compared to other splenic masses (100). Yoshikawa et al. applied miR-214/5AE, a synthetic miR-214, to mice with canine angiosarcoma. They found that synthetic miR-214, through the overexpression of cleaved caspase-3 and *p53*, can induce apoptosis and inhibit cell proliferation (101). On the other hand, Except for glioblastoma, bladder cancer, and angiosarcoma, miR-210 is up-regulated in nearly all of the cancer types that have been studied (102).

miR-497-5p has emerged as a tumor suppressor in angiosarcoma, inhibiting cell proliferation, metastasis, and viability (82). Furthermore, Benton et al. discovered that miR-497 overexpression drastically decreased cell migration and tumor development (82).While KCa3.1 was previously identified as a target of miR-497-5p, with studies demonstrating its ability to suppress tumor invasion by over 70% (81), recent research suggests the presence of additional targets. Specifically, Cdk6, Ccnd2, and Vat1 have been identified as potential downstream effectors of miR-497-5p in angiosarcoma, highlighting the complex regulatory network it governs (82).

Studies show that vinyl chloride exposure alters miRNA expression in rat hepatocytes. This exposure decreases the expression of tumor-suppressive miRNAs like miR-195, miR-199a, and miR-125b, while increasing miR-222, an oncogenic miRNA. These changes in miRNA levels lead to altered activity of target proteins involved in cell cycle regulation, such as cyclin D1, cyclin A, CDK6, and p27. Interestingly, unlike in the liver, serum miRNA expression remains unaffected by vinyl chloride exposures. This highlights the potential of miRNAs in liver tissue, but not serum, as early diagnostic markers for vinyl chloride-induced liver cancers (24).

Several miRNAs contribute to angiosarcoma development by regulating key cellular processes. Nakashima et al. demonstrated that miR-210 downregulation in angiosarcoma, compared to cherry angiomas, leads to increased protein levels of its targets, ephrinA3 and E2F3, both involved in cell proliferation. miR-210 primarily regulates translation, rather than transcription, of these factors, and its decrease results in reduced cell proliferation in angiosarcoma cells (22). Wang et al. also showed in their study that miR-340 inhibits angiosarcoma cell invasion and proliferation by targeting the 3’-UTR region of SIRT7 in angiosarcoma cells (21). MYC amplification, observed in both primary and secondary angiosarcomas, can also indirectly influence angiosarcoma development [88]. MYC amplification can lead to decreased expression of THBS1 through the upregulation of the miR-17-92 cluster, thereby promoting the angiogenic phenotype of the disease (103). Interestingly, FoxO1, a transcription factor, appears to play a complex role in angiosarcoma. While FoxO1 suppression leads to reduced c-MYC expression and cell proliferation, it also results in decreased miR-34 expression. FoxO1 binding to DNA is crucial for transcribing genes involved in c-MYC signaling and miR-34c expression. Mutations in the FoxO1 protein (FoxO1Ser218) can impair its ability to activate miR-34, leading to MYC overexpression and increased cell proliferation in primary cutaneous angiosarcomas (54).

## 4 MicroRNAs and other cardiovascular system-related tumors

While this paper has predominantly discussed the role of miRNAs in angiosarcomas, emerging evidence indicates their crucial regulatory role in several prevalent cardiovascular malignancies. The following are six instances of miRNAs and the functions they play in cancers of the cardiovascular system:

1. **miR-485-5p (7q22.1):** miR-485-5p is an important microRNA that has shown roles in tumors and the cardiovascular system. It affects how the cardiovascular system looks and works. Heart fibroblast activation can be enhanced by miR-485-5p deficiency (104).
2. **Maternally expressed gene 3 (MEG3):** MEG3 is an lncRNA that plays a role in various events, including apoptosis, inflammation, oxidative stress, and endoplasmic reticulum stress, directly or through competitive binding with miRNA. Cardiovascular disorders and cancers are among the many conditions it has a role (105).
3. **Circular RNAs:** Single-stranded, covalently closed non-coding RNA molecules called circRNAs are being studied as gene expression epigenetic controllers (13). They are class of RNA molecules formed by the back-splicing of precursor messenger RNAs. Recently, circRNAs have garnered interest for their potential to regulate gene expression via sponging microRNAs, interacting with RNA-binding proteins, controlling transcription and splicing, and protein translation (106). In addition to being involved in a wide range of biological processes, they have strong associations with several illnesses, including cancer, diabetes, disorders of the neurological system, and cardiovascular disease. They have several potential uses, such as biomarkers, RNA-binding protein interactions, and miRNA sponges (107). For instance, Nakashima et al. found that circ_0024169/CUL5 ratio may be a diagnostic biomarker for vascular malignancies, whereas circ_0024169 levels may be a prognosis marker for angiosarcoma (108).
4. **Ferroptosis-related genes:** Ferroptosis is a kind of cell death not caused by apoptosis and is linked to several cardiovascular disorders. The regulatory network of mRNA-miRNA interactions including genes associated with ferroptosis, plays a crucial role in the occurrence of myocardial ischemia-reperfusion damage (109).
5. **Nonvalvular Endovascular Infections:** They typically occur in conjunction with intravascular devices and prosthetic material. They may include primary cardiac tumors, such as myxomas. Common risk factors encompass cardiovascular disease, diabetes mellitus, and cancer (110).
6. **KIAA1429 (m6A Methyltransferase):** m6A methyltransferase is essential for RNA metabolism and is implicated in several biological processes, such as cell proliferation, differentiation, and death. The dysregulation of KIAA1429 has been linked to a range of disorders, including cardiovascular diseases and cancer (111).

The following sections will delve into the engagement of miRNAs in these diverse cardiovascular tumors.

### 4.1 Rhabdomyosarcoma

Numerous miRNAs intricately regulate rhabdomyosarcoma, with notable tumor suppressors including miR-206, miR-1, miR-29, and miR-26a (112–126). A conspicuous reduction in miR-206 levels is observed within rhabdomyosarcoma tissues and cells. The elevated presence of miR-206 induces myogenic differentiation and impedes tumor growth by targeting essential genes like c-Met, PAX3, and G6PD (114–116, 118–122). By suppressing the expression of c-Met and PAX3, miR-1 also acts as a tumor suppressor, resulting in cell cycle arrest and autophagic cell death (115, 117, 118, 124). Inhibiting tumor growth, miR-29 targets explicitly cyclin D2 and E2F7 (112, 118, 126), and miR-26a, downregulated in this context, exerts tumor-suppressive effects by reducing EZH2 expression (113, 125). These miRNAs impede proliferation, induce apoptosis and differentiation, and target pivotal oncogenes, suppressing rhabdomyosarcoma progression.

Emerging evidence suggests a multifaceted tumor-suppressive role for miR-206 in rhabdomyosarcoma, making it a promising candidate for future therapeutic development (114–116, 118–122). Restoration of miR-206 expression holds therapeutic potential, warranting further investigation into targeted delivery methods for rhabdomyosarcoma treatment (114). Hence, miRNAs assume a crucial role in rhabdomyosarcoma through their diverse mechanisms by functioning as tumor suppressors.

### 4.2 Hemangioma

miRNAs play a diverse regulatory role in hemangioma progression by targeting key signaling pathways. Notably, miR-130a exhibits a consistent upregulation in hemangioma tissues and cell lines compared to normal controls. Functional studies revealed that inhibiting miR-130a significantly reduces proliferation and tumor growth by targeting TFPI2 and modulating the FAK/PI3K/Rac1/Mdm2 pathway, highlighting its potential as a therapeutic target (127). Propranolol, a beta-blocker medication, has been shown to decrease miR-382 levels in hemangiomas, leading to reduced tumor progression through activation of the PTEN/AKT/mTOR pathway (128). DNA methylation alterations are also implicated in hemangioma development. The combination of DNMT3A inhibition and miR-206 overexpression has been shown to promote extracellular matrix deposition and impede malignant transformation of hemangioma endothelial cells, suggesting a potential therapeutic approach (129). Beyond protein-coding genes, miRNAs can interact with long non-coding RNAs (lncRNAs) to regulate hemangioma development. An inverse correlation exists between the expression of lncRNA NEAT1 and miR-361-5p in hemangioma tissues. Knockdown of NEAT1 restrains hemangioma endothelial cell proliferation and migration by sequestering miR-361-5p and modulating VEGFA expression (130). Similarly, miR-125a-3p acts as a negative regulator of hemangioma endothelial cells by interacting with the oncogenic lncRNA CASC9, controlling proliferation, migration, and invasion (131). Signaling pathways crucial for development and differentiation are also miRNA targets in hemangioma. Suppression of miR-203, a regulator of Notch signaling, promotes proliferation and inhibits apoptosis in hemangioma endothelial cells (132). Furthermore, reduced levels of miR-497-5p enhance proliferation and diminish ferroptosis (iron-dependent cell death) through modulation of the Notch2 pathway (133). Therefore, various microRNAs intricately regulate hemangioma progression by interacting with different genes and pathways. MiR-130a, in particular, stands out due to its consistent upregulation across hemangiomas, making it a promising candidate for therapeutic targeting.

### 4.3 Cardiac myxoma

In cardiac myxoma, miR-217 acts as a tumor suppressor by negatively regulating the oncogenic IL-6 gene. Therapeutic modulation of miR-217 levels may provide a potential avenue for managing this primary heart tumor (134). **Table 1** summarizes the mechanisms by which various miRNAs affect distinct types of cardiovascular tumors.

**Table 1:** Action mechanism of various miRNAs on distinct types of cardiovascular tumors.

Cardiovascular system-related tumor	MicroRNA expression status	Influence/mechanism(s)	Model	Ref.
Angiosarcoma	miR-541-3p/654-5p/1-3p/128-3p/485-5p/381-3p/370-3p/377-3p/193a-3p/153-3p/504-5p (Up-regulated)	• Integration of expression profiles with miRNA expression data indicated probable important miRNA-gene regulator connections for each sarcoma subtype. • The anticipated targets HMCN1, NKX2-2, SCNN1G, and SOX2 were increased in Ewing’s sarcoma and fibromatosis samples, where miR-182-5p is downregulated.	*ex vivo*	(95)
	miR-515-5p/517a/517c/518b/519a/522 (Overexpressed)	Overall, S-MED serves as a valuable resource for understanding the miRNA expression patterns and potential mechanisms involved in angiosarcoma.	*ex vivo*	(135)
	miR-126 (oncogene), (Up-regulated)	• TP53 loss is selective in pCAS, while myc amplification/overexpression is indicative of sCAS. Both pCAS and sCAS exhibit considerable molecular heterogeneity. • Affected genes and their molecular regulators, including miRNAs, may serve as future drug targets.	*in situ*	(18)
	miR-452/494-3p/214-3p/543/497-5p (Up-regulated)	• Serum microRNAs differed between dogs with splenic tumors and healthy dogs with histologically normal spleens.	*in vivo*	(100)
	miR-497 (Tumor suppressor), (Down-regulated)	• miR-497-5p (miR-497) significantly suppresses cell viability in AS. • miR-497 overexpression leads to reduced cell migration and tumor formation in AS and directly leads to reduced cell migration and tumor formation in AS, and can regulate VAT1, suggesting that VAT1 may promote cell migration in AS • miR-497 mimic transfection increases apoptosis in AS and hemangioendothelioma cell lines.	*in vitro* and *in vivo*	(82)
	miR-195/199a/125b (Tumor suppressor), (Down-regulated) miR-222 (Tumor suppressor), (Down-regulated initially, then Up-regulated)	• Under Vinyl chloride (VC) exposure, hepatocellular carcinoma and angiosarcoma miRNA-222, miRNA-199a, miRNA-195, and miRNA-125b levels varied dynamically. MiRNA-222 first reduced and then grew, while miRNA-199a, 195, and 125b increased and then declined. Cell cycle distribution and expression of cell cycle-related proteins (p27, cyclinA, cyclinD1, and CDK6) changed. • These miRNAs’ serum levels remained unchanged. • Cell cycle-related proteins can be deregulated by VC-induced dynamic changes in miR-222, miR-199a, miR-195, and miR-125b. These miRNAs may be early indicators for VC-induced cancer.	*in vivo*	(24)
	miR-340 (Tumor suppressor), (Down-regulated)	• miR-340 expression was considerably lower in angiosarcoma than controls. • Overexpression of miR-340 decreased angiosarcoma cell proliferation and invasion. • Sirtuin 7 (SIRT7) may be a miR-340 target gene. • Overexpression of SIRT7 increased angiosarcoma cell proliferation and invasion and partially reversed miR-340’s anticancer impact.	*in vitro* and *ex vivo*	(21)
	miR-210 (Tumor suppressor), (Down-regulated)	• miR-210 was downregulated *in vivo* and *in vitro* in angiosarcoma cells. • E2F3 and ephrin A3 were upregulated in angiosarcoma tumor cells. • Angiosarcoma cell numbers decreased significantly after E2F3 or ephrin A3 knockdown. • Angiosarcoma patients not significantly differed from healthy controls in serum miR-210 levels.	*in vitro* and *ex vivo*	(22)
	miR-17-92 (oncogene), (Up-regulated) miR-18a and miR-19a (Down-regulated)	• The miR-17-92 cluster was significantly elevated in MYC-amplified AS compared to AS without amplification and the control group. • In AS with MYC amplification, miR-18a and miR-19a from the miR-17-92 cluster down-regulated THBS1 and CTGF gene mRNA expression. • By inhibiting clusterin production via the TGF-b signaling pathway, the miR-17-92 cluster promotes angiogenesis. • CCN family member CTGF was downregulated in MYC-amplified AS, suggesting its role in angiogenesis suppression in this sarcoma subtype.	*In vitro* and *in situ*	(103)
	miR-34c (Tumor suppressor), (Up-regulated)	• aPKCλ phosphorylates FoxO1’s DNA binding domain, promoting endothelial cell proliferation and c-Myc expression. • Angiosarcoma cell growth and c-Myc expression decrease with aPKC inhibition.	*In vitro*, and *in vivo*	(54)
	miR-23 (Up-regulated)	• In rat and human angiosarcoma, miR-23 target genes (Ccnd1, Adam19, Plau, and Wsb1) that increase invasiveness and metastasis were enriched.	*In vitro, in vivo* and *in situ*	(90)
Canine angiosarcoma	miR-214/5AE, (synthetic) (Anti-tumor effects)	• p53, induces apoptosis, and inhibits cell proliferation	*in vivo*	(101)
Canine hemangiosarcoma	miR-214, (Tumor suppressor) (Downregulated)	• Through p53-regulated gene transcription, miR-214 restoration decreased cell proliferation and triggered apoptosis in canine hemangiosarcoma cell lines. • HSA cell lines overexpressed COP1, and knocking it down caused apoptosis and upregulated p53-regulated genes. • COP1 knockdown induced apoptosis in HSA cells by up-regulating p53-regulated gene expression, similar to the results obtained using miR-214-transfection.	*in vitro*	(83)
Cardiac myxoma (CM)	miR-217, (Tumor suppressor) (Down-regulated)	• miR-217 expression is downregulated in CM tissues and is inversely correlated with IL-6 mRNA expression. • Overexpression of miR-217 reduces primary CM cell growth and induces apoptosis. • miR-217 directly targets IL-6, as shown by dual luciferase experiments. • IL-6 downregulation by siRNA mimics miR-217’s tumor-suppressive effects in CM. • The anti-proliferative and pro-apoptotic impact of miR-217 overexpression in CM cells is reversed by IL-6 restoration.	*in situ*	(134)
Hemangioma	miR-203, (Tumor suppressor) (Down-regulated)	• Knockdown of MEG8 upregulates miR-203 expression. • HemECs’ MEG8 knockdown is reversed by MiR-203 silencing. • The MEG8 knockdown reduces JAG1 and Notch1 expression in HemECs.	*in vitro*	(132)
	miR-452 (oncogene) (Up-regulated)	• Expression higher in serum of dogs with splenic hemangiosarcoma • Increasing EGFR and Akt signaling • EGFR supports anchorage-independent growth and metastasis in canine hemangiosarcoma.	*in vivo*	(100)
	miR-497-5p, (Tumor suppressor) (Down-regulated)	• Silencing MEG8 inhibited the proliferation of HemECs, increased the level of MDA in HemECs, decreased the expression of SLC7A11 and GPX4 in HemECs, but no effect on the level of AIFM2. • Blocking miR-497-5p reversed the effects of MEG8 loss on cell viability, MDA level, and expression levels of NOTCH2, SLC7A11, and GPX4 in HemECs.	*in vitro*	(133)
	miR-361-5p, (Tumor suppressor) (Down-regulated)	• NEAT1 expression was higher in HA tissues, notably proliferative phase HAs, than in skin. MiR-361-5p expression reduced in HA tissues, especially proliferating HAs. NEAT1 functioned as a competing endogenous RNA to regulate VEGFA expression by sponging miR-361-5p. • Downregulation of NEAT1 dramatically reduced HemEC viability, PCNA expression, and migration while increased apoptosis and caspase-3 activity. • Knockdown of NEAT1 suppressed the proliferation and migration, but facilitated the apoptosis of HemECs. • NEAT1 enhanced the proliferation and migration, and decreased the apoptosis of HemECs via negatively regulating miR-361-5p.	*In vitro*	(130)
	miR-125-3p, (Tumor suppressor) (Down-regulated)	• CASC9 knockdown significantly inhibited proliferation, migration, and invasion of HDECs. CASC9 knockdown reduced cyclinD1, N-cadherin, Twist, and MMP2 translation. Also, CASC9 knockdown decreased Twist, MMP2, cyclinD1, and N-cadherin translation. • CASC9 interacts with miR-125a-3p/Nrg1 to regulate cellular functions. miR-125a-3p can reverse the effect of CASC9 on proliferation, migration, and invasion of HDECs. • CASC9 over-expression exerted the opposite effect, promoting proliferation, migration, and invasion of HDECs. • CASC9 over-expression exerted the opposite effect, promoting proliferation, migration, and invasion of HDECs.	*In vitro* and *in vivo*	(131)
Infantile Hemangioma (IH)	miR-382, (Tumor suppressor) (Up-regulated)	• miR-382 is overexpressed in IH • miR-382 downregulates PTEN • miR-382 activates AKT/mTOR signaling • Promoting IH progression	*In vitro*	(128)
	miR-206, (Tumor suppressor) (Down-regulated)	• MiR-206 downregulated in proliferative hemangioma suppressed HemEC malignancy via modulating ECM-related genes. • *In vitro* and *in vivo* research showed that miR-206 increases ECM accumulation by targeting DNMT3A, preventing HemEC malignancy and alleviating IH. • Overexpression of DNMT3A prevented miR-206 mimic from inhibiting HemECs and regulating ECM.	*In vitro* and *in vitro*	(129)
Rhabdomyosarcoma	miR-206, (Tumor suppressor) (Down-regulated)	• miR-206 modulates over 700 genes to restore differentiation in RMS cells. • In ERMS cells, silencing G6PD, a miR-206 target, inhibits proliferation and soft agar growth. G6PD overexpression does not affect miR-206’s pro-differentiation impact. • DCA improves miR-206-induced RMS cell growth inhibition and maintains it after de-induction. This shows combining differentiation-inducing and metabolism-directed techniques may be advantageous.	*in vitro*	(114, 120)
	miR-1/-206/-29 (Tumor suppressor) (Down-regulated)	• Cell cycle gene CCND2 is regulated by miR-1, -206, and -29. • Similar to CCND2, miR-29 targets E2F7, a cell cycle regulator. • Cell function study demonstrates that miR-29 overexpression downregulates several cell cycle genes, promotes partial G1 arrest, and decreases cell proliferation.	*in vitro* and *ex vivo*	(118)
	miR-26a (Tumor suppressor) (Down-regulated)	• This study reveals deregulation of miR-26a and Ezh2 in rhabdomyosarcoma samples and cell lines, suggesting their potential involvement in rhabdomyosarcoma pathogenesis.	*in vitro*	(113)
	miR-1/206	• miR-1/206 decreased c-Met expression in rhabdomyosarcoma and may be a strong tumor suppressor. • Inhibition of miR-1/206 may cause abnormal cell growth and migration, causing rhabdomyosarcoma.	*in vitro* and *ex vivo*	(136)
	miR-29 (Tumor suppressor) (Down-regulated)	• miR-29a suppressed subcutaneous xenograft carcinogenesis in nude mice and GEFT mRNA and protein expression in transplanted tumors.	*in vitro* and *ex vivo*	(126)
	miR-26a (Tumor suppressor) (Down-regulated)	• Plasma levels of muscle-specific miR-206 were substantially higher in RMS patients than healthy donors. • In an independent larger cohort of patients (validation set), digital droplet PCR detected reduced miR-26a and miR-30b/c levels.	*ex vivo*	(125)

AS, Angiosarcoma; CAS, Cutaneous angiosarcoma; CM, Cardiac myxoma; DNMT3A, DNA Methyltransferase 3A; IH, Infantile Hemangioma; MDA, malondialdehyde; pCAS, spontaneous cutaneous angiosarcoma.

## 5 Future directions and conclusion

The current review highlights the diverse roles of miRNAs in angiosarcoma and other cardiovascular tumors. Despite the promising findings, it is evident that further research is imperative before miRNAs can be seamlessly integrated into clinical practice as diagnostic or prognostic markers or therapeutic targets. Future investigations should extend beyond angiosarcomas to explore miRNA expression and interactions in other cardiovascular tumors that have received less attention. A crucial aspect is understanding how miRNAs contribute to the molecular biology of these tumors and comparing their potential benefits or drawbacks against current diagnostic and prognostic approaches.

As previously discussed, examples such as miR-217 suppressing the carcinogenic IL-6 gene in cardiac myxoma and miR-1 inhibiting c-Met and PAX3 to suppress tumor growth in rhabdomyosarcoma showcase the diverse roles of miRNAs in different tumors. Additionally, miR-340 targeting SIRT7’s 3’-UTR to decrease angiosarcoma cell invasion and proliferation demonstrates the specificity of miRNA actions. Nonetheless, the levels of miR-214 were significantly higher in angiosarcoma tissues compared to non-angiosarcoma tissues. Elucidating the precise mechanisms, interactions, and potential side effects of therapeutic tools targeting miRNAs is essential for translating these findings into clinical applications. This knowledge will provide the foundation for designing randomized control trials and facilitating the clinical application of miRNA-based interventions. Despite the progress made, there remains a significant gap in our understanding before miRNAs can be considered reliable diagnostic or prognostic markers or plausible therapeutic targets in clinical settings.

Overall, this review emphasizes the potential of miRNAs as diagnostic, prognostic, and therapeutic tools in cardiovascular tumors, including angiosarcoma, a challenging soft-tissue sarcoma. Furthermore, it explores the potential contributions of miRNAs to other cardiovascular tumors, proposing their utility as diagnostic, prognostic markers, and therapeutic targets. Although the area is progressing, our comprehension of the complex function of miRNAs in cardiovascular malignancies is still in its nascent phase, requiring further research to reveal their therapeutic usefulness and underlying processes.

## Author contributions

AM: Writing – original draft, Validation. AA: Supervision,Writing – original draft. NE: Writing – original draft. SR: Writing – original draft. NR: Writing – original draft. SD: Writing – original draft, Visualization. FF: Supervision, Writing – original draft. ZN: Writing – original draft. RA: Writing – review & editing. HM: Supervision, Writing – review & editing.
